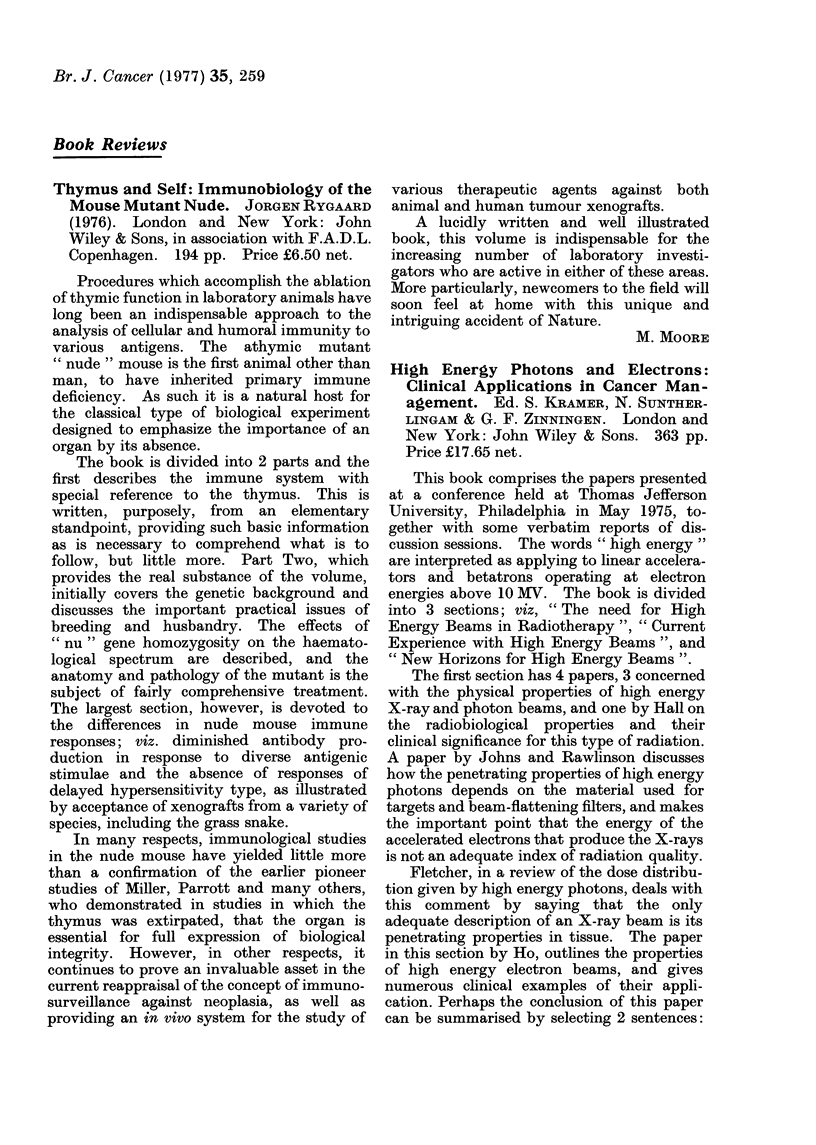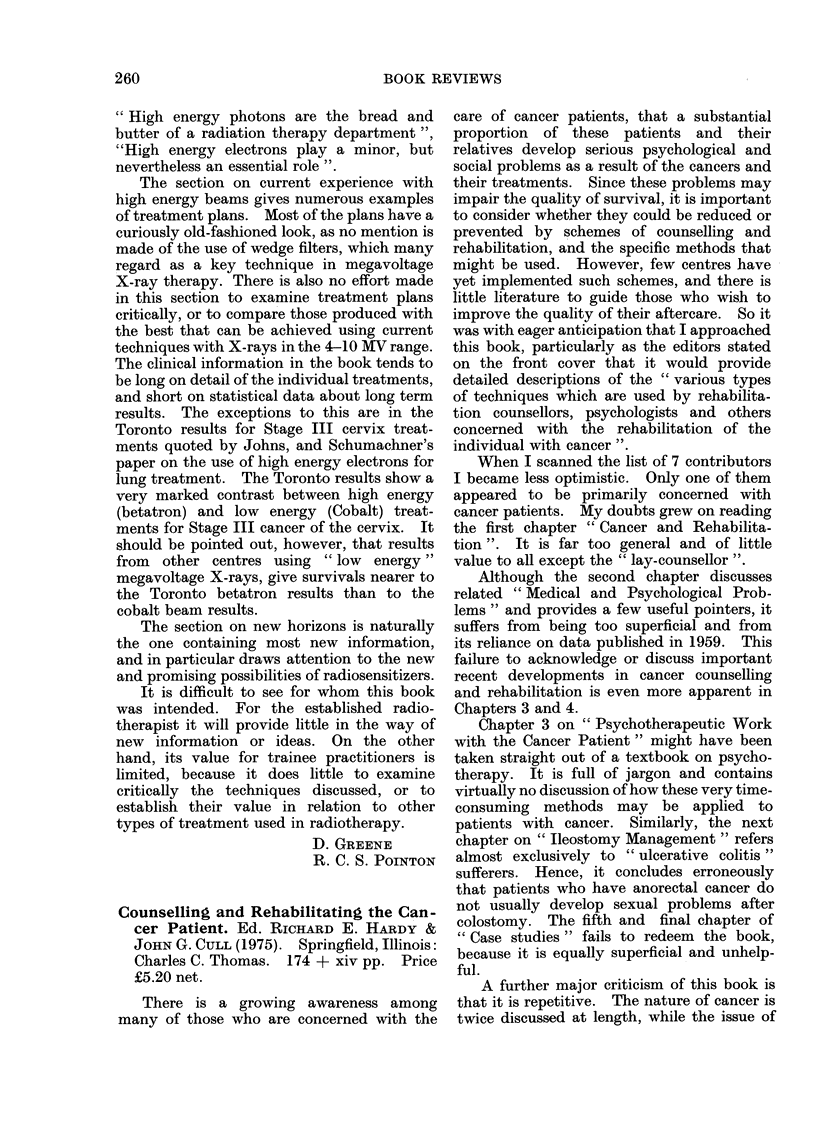# High Energy Photons and Electrons: Clinical Applications in Cancer Management

**Published:** 1977-02

**Authors:** D. Greene, R. C. S. Pointon


**High Energy Photons and Electrons: Clinical Applications in Cancer Management.** Ed. S. KRAMER, N. SUNTHERLINGAM & G. F. ZINNINGEN. London and New York: John Wiley & Sons. 363 pp. Price £17.65 net.

This book comprises the papers presented at a conference held at Thomas Jefferson University, Philadelphia in May 1975, together with some verbatim reports of discussion sessions. The words " high energy " are interpreted as applying to linear accelerators and betatrons operating at electron energies above 10 MV. The book is divided into 3 sections; *viz,* " The need for High Energy Beams in Radiotherapy ", " Current Experience with High Energy Beams ", and " New Horizons for High Energy Beams ".

The first section has 4 papers, 3 concerned with the physical properties of high energy X-ray and photon beams, and one by Hall on the radiobiological properties and their clinical significance for this type of radiation. A paper by Johns and Rawlinson discusses how the penetrating properties of high energy photons depends on the material used for targets and beam-flattening filters, and makes the important point that the energy of the accelerated electrons that produce the X-rays is not an adequate index of radiation quality.

Fletcher, in a review of the dose distribution given by high energy photons, deals with this comment by saying that the only adequate description of an X-ray beam is its penetrating properties in tissue. The paper in this section by Ho, outlines the properties of high energy electron beams, and gives numerous clinical examples of their application. Perhaps the conclusion of this paper can be summarised by selecting 2 sentences:

" High energy photons are the bread and butter of a radiation therapy department ", "High energy electrons play a minor, but nevertheless an essential role ".

The section on current experience with high energy beams gives numerous examples of treatment plans. Most of the plans have a curiously old-fashioned look, as no mention is made of the use of wedge filters, which many regard as a key technique in megavoltage X-ray therapy. There is also no effort made in this section to examine treatment plans critically, or to compare those produced with the best that can be achieved using current techniques with X-rays in the 4–10 MV range. The clinical information in the book tends to be long on detail of the individual treatments, and short on statistical data about long term results. The exceptions to this are in the Toronto results for Stage III cervix treatments quoted by Johns, and Schumachner's paper on the use of high energy electrons for lung treatment. The Toronto results show a very marked contrast between high energy (betatron) and low energy (Cobalt) treatments for Stage III cancer of the cervix. It should be pointed out, however, that results from other centres using " low energy " megavoltage X-rays, give survivals nearer to the Toronto betatron results than to the cobalt beam results.

The section on new horizons is naturally the one containing most new information, and in particular draws attention to the new and promising possibilities of radiosensitizers.

It is difficult to see for whom this book was intended. For the established radiotherapist it will provide little in the way of new information or ideas. On the other hand, its value for trainee practitioners is limited, because it does little to examine critically the techniques discussed, or to establish their value in relation to other types of treatment used in radiotherapy.

D. GREENE
R. C. S. POINTON